# Area-Time-Efficient Secure Comb Scalar Multiplication Architecture Based on Recoding

**DOI:** 10.3390/mi15101238

**Published:** 2024-10-07

**Authors:** Zhantao Zhang, Weijiang Wang, Jingqi Zhang, Xiang He, Mingzhi Ma, Shiwei Ren, Hua Dang

**Affiliations:** 1 School of Integrated Circuits and Electronics, Beijing Institute of Technology (BIT), Beijing 100081, China; 3120231354@bit.edu.cn (Z.Z.); wangweijiang@bit.edu.cn (W.W.); zhangjq@bit.edu.cn (J.Z.); 3220221592@bit.edu.cn (X.H.); bitmilesma@gmail.com (M.M.); renshiwei@bit.edu.cn (S.R.); 2 Chongqing Institute of Microelectronics and Microsystems, Beijing Institute of Technology, Chongqing 404100, China

**Keywords:** elliptic curve cryptography (ECC), elliptic curve scalar multiplication (ECSM), field-programmable gate array (FPGA), prime field (GF), sample power analysis (SPA)

## Abstract

With the development of mobile communication, digital signatures with low latency, low area, and high security are in increasing demand. Elliptic curve cryptography (ECC) is widely used because of its security and lightweight. Elliptic curve scalar multiplication (ECSM) is the basic arithmetic in ECC. Based on this background information, we propose our own research objectives. In this paper, a low-latency and low-area ECSM architecture based on the comb algorithm is proposed. The detailed methodology is as follows. The recoding-k algorithm and randomization-Z algorithm are used to improve security, which can resist sample power analysis (SPA) and differential power analysis (DPA). A low-area multi-functional architecture for comb is proposed, which takes into account different stages of the comb algorithm. Based on this, the data dependency is considered and the comb architecture is optimized to achieve a uniform and efficient execution pattern. The interleaved modular multiplication algorithm and modified binary inverse algorithm are used to achieve short clock cycle delay and high frequency while taking into account the need for a low area. The proposed architecture has been implemented on Xilinx Virtex-7 series FPGA to perform ECSM on 256-bits prime field GF(p). In the hardware architecture with only 7351 slices of resource usage, a single ECSM only takes 0.74 ms, resulting in an area-time product (ATP) of 5.41. The implementation results show that our design can compete with the existing state-of-the-art engineering in terms of performance and has higher security. Our design is suitable for computing scenarios where security and computing speed are required. The implementation of the overall architecture is of great significance and inspiration to the research community.

## 1. Introduction

In this section, we provide a comprehensive background on the topic, which will help in understanding the context and importance of the research. We will then review the related work to explain the advancements and limitations in the current research landscape. Finally, we will present the motivation and outline the main contributions.

### 1.1. Background

The rapid development of mobile communication, artificial intelligence, and mobile ultra-wideband has led to a rapid increase in data transmission density and frequency [1]. At the same time, as convenience increases, the security of personal data is also challenged [2]. In these scenarios, the security of data encryption is critical [3]. Therefore, people pay more and more attention to encryption algorithms. Encryption algorithms are divided into the symmetric encryption algorithm and the public key algorithm. In the application scenarios of digital signature and key negotiation, the public key algorithm can support the secure communication of a large number of users and protect the privacy of both parties, so it is widely used [4].

The classic algorithms of public key algorithms are Rivest–Shamir–Adleman (RSA) and elliptic curve cryptography. RSA was invented by Rivest and Adleman [5]. RSA typically requires long keys, making it difficult to improve computational efficiency. ECC originated in the 1980s and was independently proposed by Neal Koblitz and Victor S. Miller [6,7]. The implementation principle of ECC is based on the complexity and irreversibility of the elliptic curve discrete logarithm problem (ECDLP). The main advantage of ECC is the shorter key length required to guarantee the same security [8]. For example, a 256-bit ECC key has the same security as a 3072-bit RSA key [9,10]. This not only reduces the need for storage and transmission but also improves computing efficiency. ECC is now widely used in various encryption protocols and systems, such as key exchange protocols in blockchain technology, digital signature algorithms, public and private key generation, and transaction signatures.

The key core operation in ECC is elliptic curve scalar multiplication. ECSM is both the ECC security core and the ECC performance core. Through the calculation of ECSM, ECDLP is introduced. In this way, the mathematical security of the algorithm is guaranteed. ECSM is also the part of ECC that limits performance and is the most time-consuming operation that needs to be optimized. The implementation principle of ECSM is to iterate through elliptic curve point addition (ECPA) elliptic curve point doubling (ECPD) [11]. Among them, ECPD and ECPA are implemented through finite field operations.

Depending on the finite field, ECSM can be implemented in both the prime field and the binary field [12,13]. Relatively mature attack methods and complexity analysis for ECSM in the prime field already exist. In contrast, there is less research on attacks on binary-field ECSM, and some attack methods may be more effective against binary-field ECSM. In addition, ECC encryption implemented in binary fields is more vulnerable to attack by specific algorithms [14,15]. Prime-field ECSM is not only safer than binary field, but also widely used. Many international encryption standards adopt prime field ECC implementation, such as NIST standard, SECG standard, ISO/IEC standard [16,17,18].

Prime-field ECSM can be carried out on a generic prime or some special prime. The curve on a generic prime such as secp256r1 has broad standard support and compatibility benefits and is widely adopted by many protocols and systems, ensuring good interoperability with existing systems. Although curves on a special prime such as the Montgomery curve, Edwards curve, and Curve25519 curve perform well in some aspects of performance and security, their compatibility problems and standardization are relatively low, making them difficult to use in some application scenarios [19]. Therefore, to ensure reliability and compatibility, we choose to use a universal curve.

Because of the complexity of ECSM calculation, hardware is usually used to accelerate the calculation during the implementation. Hardware acceleration platforms are mainly divided into FPGA and application specific integrated circuit (ASIC). Compared with ASIC, FPGA implementation of ECC has many advantages. First, FPGA has a high degree of parallel processing capability, which can significantly speed up the execution of ECSM. Second, the flexibility and reconfigurability of FPGA allows it to flexibly adapt to different optimization algorithms. FPGA allows hardware implementations of ECC algorithms to be customized for specific application scenarios, optimizing performance and resource usage.

When using ASIC or FPGA to implement ECSM, it is usually necessary to deal with sensitive information, such as private keys (pks). Therefore, in the calculation process, the hardware computing platform will inevitably disclose some information through the way of side channels, such as power consumption differences, electromagnetic radiation, and so on [20]. Therefore, in the ECSM implementation process, it is necessary to consider not only the security of mathematical perspective, but also the security of physical implementation, that is, to consider the side-channel attack (SCA) defense [21]. The ability to resist SCA is fundamental to maintaining the confidentiality and integrity of encryption operations. Only the implementation of SCA resistance can ensure the security of ECSM, and thus the security of the entire ECC encryption protocol.

### 1.2. Related Work

First, the implementation process of ECSM can be summarized into two methods. The first is the serial calculation method and the second is the parallel calculation method. The serial-based ECSM algorithm iterates over all bits of the scalar in turn. Erdem [22] investigates the numeric serial implementation of the Montgomery algorithm for large integers. Mehrabi [11] proposed an ECC kernel hardware based on the residual number system (RNS), support fast elliptic curve point addition, elliptic curve point doubling, and elliptic curve point triple (ECPT). The serial ECSM algorithm requires a relatively large number of cycles. This method of calculation often leads to the lengthening of the calculation cycles or the need for area to compensate. Therefore, many scholars are also studying the ECSM algorithm based on parallel calculation.

The parallel ECSM algorithm often brings a pre-calculation burden. Javeed [23] implements ECSM in the general prime number field GF(p) based on the efficient $radix-2^{3}$ parallel GF(p) multiplier. Cui et al. [24] implemented iterative digit–digit Montgomery multiplication (IDDMM) and optimized the elliptic curve point operation data flow architecture based on parallel hardware. Salarifard [25] presents two low-complexity (LC) and low-latency (LL) architectures based on the comb algorithm at $GF(2^{m})$. The parallel ECSM algorithm sometimes also causes the area of the control circuit and the storage circuit to increase, resulting in the overall circuit becoming large. How to balance pre-calculation and the main loop calculation burden needs to be further studied.

As well as the macroscopic ECSM implementation method, coding optimization is also being gradually paid attention to, which is used to improve the operation speed and reduce the resource consumption. Shylashree et al. [26] proposed a ternary coding method and used pre-calculation to speed up calculation. Phalakarn [27] introduces an optimal representation of scalar point multiplication on parallel elliptic curves from right to left. The superiority of non-adjacent form (NAF) coding is verified by a simplified Robert model. Khleborodov [28] further proposed the window non-adjacent form (ω-NAF) of the scalar representation method. The computational complexity theorem of the algorithm is presented and proved. Not using proper coding often results in unnecessary computational complexity or reduced security. The coding methods are complementary to the computational methods of ECSM. The performance and security of ECSM can be improved effectively by using appropriate coding methods.

Some studies have optimized specific classes of curves to improve the performance and security of ECSM architectures. Many standards publish curves that are currently recognized as safe. For example, the IEEE1363 standard and NIST standard. Salarifard [29] and Sasdrich [30], respectively, optimized and realized the safe and lightweight ECC curve, which is called curve25519. De [31] implements an optimized acceleration on the Edwards curve. Bisheh-Niasar [32] implements the best implementation of the area-time product on the Ed448 curve. Islam [33] proposes a high-performance ECC processor, which is implemented on Edwards25519. Optimizations for specific curves often result in better performance at the accompanying cost of reduced hardware flexibility and portability.

Besides improvements to ECC itself, many scholars have also focused on the design of FPGA to resist the side-channel attack. Brier [34] overcomes the side-channel attack on Weierstra ß elliptic curve. Sasdrich [30] implements resistance against a mix of side-channel attack to impede simple and differential power analysis on curve25519. In the ECSM implementation, the irreversibility of the algorithm itself is necessary, and security against the side-channel attack should not be ignored.

In general, the current serial ECSM algorithm is simple but has a long computing cycle, low efficiency, and high hardware resource demand. The parallel ECSM algorithm is efficient, but pre-calculation is heavy, which will increase the area of the control circuit and the memory circuit and make the circuit complicated. Coding optimization can improve computing speed and resource utilization, but improper selection will increase computational complexity and reduce security. Optimization for specific curves improves performance but reduces hardware flexibility and portability. Although some designs have made progress against anti-side-channel attacks, some implementations are still vulnerable to SCA.

### 1.3. Motivation and Contribution

Because many ECSM designs are implemented on specific curves, there is no hardware flexibility and portability. To solve this problem, we implement an ECSM architecture on the prime field for the universal simplified Weierstra elliptic curve in Jacobi coordinate. Because of the large number of ECSM calculations in ECC, it takes more time for ECSM to scan all bits serially. In order to solve this problem, this paper adopts the algorithm of ECSM scanning bits in parallel. Many current ECSM architectures neglect defense against side-channel attacks. In order to realize the security of ECSM computing, we are inspired to carry out corresponding hardware design and scalar coding design for side-channel attacks. In general, this paper gives consideration to computing security and provides a solution for computing scenarios that pursue computing speed but have limited resources.

The main contributions of this paper are as follows:

1. Improve the security of the ECSM architecture. Using the comb-4 algorithm avoids possible sample power analysis. The recoding-k algorithm is used to overcome the potential zero analysis attacks. Avoid redundant operations, thereby increasing security against fault injection attack. The randomization-Z algorithm is used to improve the ability of resisting differential power analysis.
2. Improve the computing speed of the ECSM architecture. Multiple scanned bits are calculated synchronously to reduce the number of main cycles. Combining the calculation cycles of the main cycle and pre-calculation, the folding times are discussed.
3. Reduce resource footprint of the ECSM architecture. In this ECSM architecture design, a multi-functional calculation processing unit that can be reused in the main cycle phase and pre-calculation phase is proposed.
4. Optimize the hardware structure. The multiplications in the main loop are interleaved by analyzing the data dependencies. Carry lookahead adder with small bit width is used in modular multiplication circuits. Thus, the working frequency of the whole system is improved, and the calculation time of ECSM is shortened.

The rest of this paper is organized as follows. Section 2 presents background knowledge. The proposed scheme for ECSM include recoding-k algorithm and comb-4 algorithm are introduced in Section 3. The optimized hardware scheduling scheme and hardware architecture are shown in Section 4. Section 5 analyzes the data dependencies and timing. In Section 6, experiment results and comparisons with existing designs are given. Finally, Section 7 is conclusion of this paper.

## 2. Preliminary

### 2.1. Finite Field Arithmetics

A finite field, also known as a Galois field, is an algebraic structure containing a finite number of elements, in which addition, subtraction, multiplication, and division operations are defined. Finite fields have important applications in modern cryptography and coding theory, especially in ECC [12].

The basic definition of a finite field is shown below. Definition of a finite field: A finite field $GF(p^{n})$ is a field containing $\left(p^{n}\right)$ elements, where *p* is a prime number and *n* is a positive integer. When n=1, the field is called a prime field, denoted as GF(p); when (n>1), it is an extended field, denoted as $GF(p^{n})$. This article implements ECC in the prime field.

The following are the specific implementations of these operation arithmetics in the prime field GF(p):

- modular addition and modular subtraction: In GF(p), elements can be considered as a set of 0,1,⋯,p−1. Addition and subtraction are achieved through modular operations. For example, for a,b∈GF(p), there are
  (1) a±b≡(a±b)modp
- modular multiplication (MM): Similar to addition, multiplication is also achieved through modular operations. For example, for a,b∈GF(p), there are
  (2) a·b≡(a·b)modp
- modular division (INV): In a finite field, division is achieved by multiplying by the inverse element. For a,b∈GF(p) and b≠0, calculating a/b is equivalent to calculating $a\cdot b^{-1}$. Here, $b^{-1}$ is the multiplication inverse of b∈GF(p), which can be obtained by the extending Euclidean algorithm.

### 2.2. ECPA and ECPD on Elliptic Curve

Elliptic curves are a type of algebraic curve with rich structures and applications, especially in cryptography and number theory, where they have important applications. A typical equation form for elliptic curves is

(3) $$y^{2}=x^{3}+ax+b$$

where a,b is a constant.

To ensure that Equation (3) defines a true elliptic curve, its discriminant is required to be non-zero. For the given equation form, the discriminant is

(4) $$\Delta =-16(4a^{3}+27b^{2})$$

In order for the curve to be non-singular, Δ≠0 is required.The collection of points on an elliptic curve includes all points that satisfy the equation (x,y) and infinity points (O), known as “zeros” or “infinity points”.

The point operation on an elliptic curve defines an addition structure, such that the set of points forms an Abelian group. Basic operations include point addition and point multiplication.

- Point addition: Take two points on a curve $P=(x_{1},y_{1})$ and $Q=(x_{2},y_{2})$. Their sum $R(x_{3},y_{3})$, which is P+Q(P≠Q), can be calculated using the following calculation formula:
  (5) $$\left\{\begin{array}{c} x_{3}=\lambda^{2}-x_{1}-x_{2}\\ y_{3}=\lambda \left(x_{1}-x_{3}\right)-y_{1} \end{array}\right.$$
  The slope λ is
  (6) $$\lambda =\frac{y_{2}-y_{1}}{x_{2}-x_{1}}$$
- Point double: If P=Q, then the calculation formula for $R(x_{4},y_{4})$, which is 2P, is as follows:
  (7) $$\left\{\begin{array}{c} x_{4}=\lambda^{2}-2x_{1}\\ y_{4}=\lambda \left(x_{1}-x_{3}\right)-y_{1} \end{array}\right.$$
  The slope λ is
  (8) $$\lambda =\frac{3x_{1}^{2}-3}{2y_{1}}$$

### 2.3. Coordinate Transformation

In ECC, two common coordinate representation methods are affine coordinate and Jacobian coordinate. Affine coordinate is the most intuitive representation method on elliptic curves. A point *P* is represented as (x,y) in an affine coordinate system, directly satisfying the elliptic curve equation. Jacobian coordinate is a homogeneous coordinate representation of points on an elliptic curve. In the Jacobian coordinate system, a point *P* is represented as (X,Y,Z) and corresponds to an affine coordinate (x,y), where

(9) $$x=\frac{X}{Z^{2}},y=\frac{Y}{Z^{3}}$$

In the Jacobian coordinate system, the elliptic curve Equation (3) is transformed into

(10) $$Y^{2}=X^{3}-aXZ^{4}+bZ^{6}$$

The main reason for choosing Jacobian coordinate in ECC is computational efficiency. In affine coordinate, calculating point addition and point multiplication involve division operations, which are costly in a finite field. In Jacobian coordinate, all calculations are completed through addition, subtraction, and multiplication, avoiding costly division.

The basic operation process of ECSM is composed of ECPA and ECPD. In Jacobian coordinate, the formulas of ECPA and ECPD are as follows [35]:

- ECPD:
  $P=\left(X,Y,Z\right),P_{3}=2P=\left(X_{3},Y_{3},Z_{3}\right),$P≠−P.
  $$A=X^{2},\;\;B=Y^{2},\;\;C=B^{2},\;\;D=Z^{2},$$
  $$E=2\left({\left(X+B\right)}^{2}-A-C\right),F=3A+aD^{2},G=F^{2}-2E.$$
  (11) $$\left\{\begin{array}{cc} X_{3} & =G,\\ Y_{3} & =F(E-G)-8C,\\ Z_{3} & ={\left(Y+Z\right)}^{2}-B-D. \end{array}\right.$$
- ECPA:
  $P_{1}=\left(X_{1},Y_{1},Z_{1}\right),P_{2}=\left(X_{2},Y_{2},Z_{2}\right),$$P_{4}=P_{1}+P_{2}=\left(X_{4},Y_{4},Z_{4}\right),$$P_{1},P_{2}\neq \infty .$
  $$A=Z_{1}^{2},\;\;B=Z_{2}^{2},\;\;C=X_{1}B,\;D=X_{2}A,$$
  $$E=Y_{1}Z_{2}B,F=Y_{2}Z_{1}A,G=D-C,H={\left(2G\right)}^{2},$$
  I=GH,J=2(F−E),K=CH.
  (12) $$\left\{\begin{array}{cc} X_{4} & =J^{2}-I-2K,\\ Y_{4} & =J(K-X_{3})-2EI,\\ Z_{4} & =({\left(Z_{1}+Z_{2}\right)}^{2}-A-B)G. \end{array}\right.$$

In the coordinate transformation of the above formula, Equation (12) after the coordinate transformation corresponds to Equation (5) before the coordinate transformation, Equation (11) after the coordinate transformation corresponds to Equation (7) before the coordinate transformation.

## 3. The Proposed Scheme for ECSM

### 3.1. Analysis of the Reasons for Choosing ω

In the actual calculation process of ECSM, the large bit width of scalar k is considered, so the serial scan calculation will lead to the lengthening of the calculation cycle. Therefore, the comb-ω algorithm is adopted in this paper. The value of ω causes a corresponding change in the pre-calculation clock cycles and the clock cycles of the main cycle. Therefore, how to balance the calculation burden of different computing stages is very important.

At present, common ECSM calculation methods that are not based on pre-calculation include the double-and-add algorithm, always double-and-add algorithm, non-adjacent form algorithm, Montgomery Ladder algorithm, Joye’s double-add algorithm, Co-Z algorithm, and so on. The non-adjacent form algorithm and double-and-add algorithm lack the balance of ECPA and ECPD calculation, so there is a hidden danger of being attacked by power analysis. The Joye’s double-add algorithm, Montgomery Ladder algorithm, and always double-and-add algorithm achieve the balance of ECPA and ECPD calculation, and can resist power analysis attacks to a certain extent. But the principle of the always double-and-add algorithm is to insert redundant operations. The added redundancy itself does not affect the calculation of any other circuit. As a result, additional redundant computing may lead to a fault injection attack.

In the comb algorithm implemented in this paper, the recoding-k algorithm is used. In this way, the algorithm achieves uniform operation and avoids the existence of redundant operation. It can effectively resist power analysis attacks and fault injection attacks. Therefore, it can be considered that among the current common ECSM algorithms, only the Montgomery Ladder algorithm and Joye’s double-add algorithm have comparable security with this design in terms of anti-side-channel attack evaluation.

Based on the comb algorithm itself, given that the bit width of the scalar being calculated is an integer power of 2, ω tends to choose an integer multiple of 2. In the calculation of ECSM, only one pre-calculation is required for the fixed base point and the calculated bit width. As the number of ECSM calculations increases, the computational burden of the pre-calculation is spread evenly across each ECSM. The burden of pre-calculation decreases.

Consider two extreme cases of pre-calculation. In the worst case, for each base point, the ECSM is calculated only once, denoted as comb-$\omega_{\#1}$. In the best case, for each base point, an infinite number of ECSM calculations are computed, denoted as comb-$\omega_{\#\infty }$. When only one ECSM is calculated, the clock cycles calculated by ECSM are equal to the pre-calculation clock cycles plus the clock cycles of the main cycle. There is no computing advantage from pre-calculation. In the case of an infinite number of ECSM calculations, the clock cycles calculated by ECSM are equal to the clock cycles of the main cycle. The burden of pre-calculation can be considered nonexistent.

Data pairs for operating ECPA and ECPD are shown in Table 1. Among them, because the Co-Z algorithm optimizes ECPD and ECPA, it adopts a more refined way to calculate. The Co-Z algorithm is scanned bit by bit and the Co-Z algorithm is performed once per round of computation, consuming 16 modular multiplications. In contrast, ordinary ECPD requires 10 modular multiplications and ordinary ECPA requires 11 modular multiplications [36]. It can be seen from the comparison that the comb method and Co-Z algorithm implemented in this paper are superior to the traditional Joye’s double-add algorithm and Montgomery Ladder algorithm. In the worst case, the algorithm implemented in this paper is better than the traditional Joye’s double-add algorithm and Montgomery Ladder algorithm. As the number of ECSM calculations increased, the advantages of our implemented approach became apparent. In comparison with the Co-Z algorithm, because the Co-Z algorithm optimizes the calculation in each cycle, the Co-Z algorithm has less computation in a single ECSM calculation. However, as the number of ECSM calculations increased and the number of ω in the comb algorithm increased, the computation cost of the comb algorithm gradually decreased.

**Figure 1:**
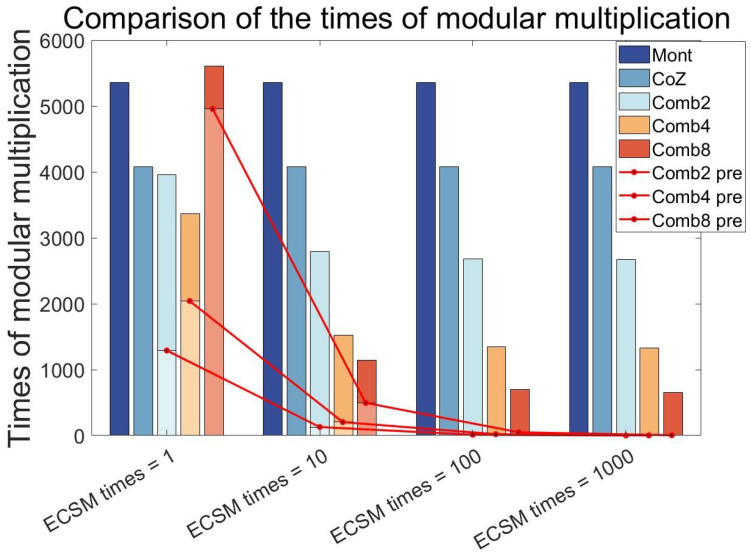
Comparison of different ω and the Co-Z algorithm and Montgomery Ladder algorithm with the increase in ECSM calculation times. In the figure, Combx_pre means the pre-calculation burden of comb-x.

In order to make a more intuitive comparison with the Co-Z algorithm, and to choose a more appropriate ω, this paper makes a more accurate comparison. This is shown in Figure 1. It can be found that when ECSM times = 1, the calculation burden of the larger ω is also large. This is because the increase in ω leads to an exponential increase in the projected burden. At ω=8, the comb’s total compute load exceeds that of the normal Montgomery Ladder algorithm. As the ECSM times increase, we can see that the pre-calculation burden is diluted. At ECSM times = 10, the comb’s advantage is already significant. However, the ω=2 calculation burden does not decrease significantly with the increase in ECSM times. This is because the computational burden of the pre-calculation and the computational advantage of halving the number of main cycles cancel each other out. To sum up, we can choose ω=4. It can not only achieve a slight advantage when ECSM times = 1, but also reduce significantly the computational burden of a single ECSM as the ECSM times increase.

### 3.2. Comb-4 Algorithm

As mentioned above, based on the balance between the pre-calculation clock cycle and the formal calculation clock cycle, this paper adopts the comb-4 algorithm to achieve 256-bit ECSM calculation. The 256 bits are divided into four groups. And then the k of the four groups of segments is synchronously scanned to reduce the clock cycles for calculating ECSM.

The specific algorithm is shown in Algorithm 1. According to Algorithm 1, we can find that in line 9 step 2, a choice is made of whether or not to perform ECPA based on whether the k values of the four different bits currently scanned are all 0. This choice can lead to an effective side-channel attack, resulting in partial leakage of the secret key. In response to the above problem, we use the recoding-k algorithm to completely avoid the situation where the *k* values of the four different bits are all 0. Specifically, in line 9 step 2 in actual ECSM, ECPA operations are performed directly without judgment, avoiding potential hazards of side-channel attacks. The recoding-k algorithm is described in detail in the next section.
**Algorithm 1** Comb-4 Algorithm**Require:** $k={\left(k_{255},\cdots ,k_{1},k_{0}\right)}_{2}$, base point $P\left(x_{P},y_{P}\right)\in GF\left(p\right),\omega =4$.**Ensure:** Q=kP.  1:**Pre-Calculation:**  2:Compute: $K_{i}P=\left[k_{i}^{\omega -1};\cdots ;k_{i}^{1};k_{i}^{0}\right]P$ for all possible $K_{i}$  3:**Calculation Stage:**  4:$d=\left\lceil \frac{m}{w}\right\rceil ,k=\left[K_{d-1},\cdots ,K_{i},\cdots ,K_{0}\right]\;\mathrm{with}\;\mathrm{zero}\;\mathrm{padding}$  5:$Q\leftarrow [k_{d-1}^{\omega -1},\cdots ,k_{d-1}^{1},k_{d-1}^{0}]P.$  6:**for** i=d−2 to 0 **do**  7:   st1: Q←ECPD(Q),  8:   **if** $[k_{i}^{w-1},\cdots ,k_{i}^{1},k_{i}^{0}]=0$ **then**  9:     st2: $Q\leftarrow ECPA(Q,\left[k_{i}^{w-1},\cdots ,k_{i}^{1},k_{i}^{0}\right]P)$10:   **end if**11:**end for**12:**Post-Processing Stage:**13:Q←Jacobian_to_affine_coordinate(Q)14:**return** $Q(x_{3},y_{3})$

### 3.3. Recoding-k Algorithm

As described above, the recoding-k algorithm can be used to enhance the comb’s ability to resist side-channel attacks. The recoding-k algorithm redefines the positive and negative of each scanned bit by introducing a new symbol bit *S*. In this way, k is recoded in such a way that the resulting comb matrix allows for the possibility that not every scanned bit will be zero.

The specific algorithm is shown in Algorithm 2. In the above algorithm, the bits scanned for the first time cannot all be 0. Therefore, in actual ECSM, according to whether the lowest position of k is 0, it is decided to add *P* or 2P before k participates in the calculation, so as to ensure that the value of the lowest position in the calculation is 1. This ensures that the first scanned bits are not all zeros. In order to eliminate the effect of this operation in the final result, we choose to subtract *P* or subtract 2P. This ensures that the results are correct. From the perspective of operation, this operation can also ensure that no matter whether the lowest level of k is 0, the operation is consistent, avoiding the possibility of a side-channel attack.
**Algorithm 2** Recoding-k algorithm**Require:** $k={\left(k_{255},\cdots ,k_{1},k_{0}\right)}_{2},\omega =4$.**Ensure:** recoding-*k*,*S*.  1:$d=\left\lceil \frac{m}{w}\right\rceil ,K_{i}=\left[k_{i}^{\omega -1};\cdots ;k_{i}^{1};k_{i}^{0}\right],$  2:$k=[K_{d-1},\cdots ,K_{i},\cdots ,K_{0}]\;\mathrm{with}\;\mathrm{zero}\;\mathrm{padding}.$  3:S[0]=1, recoding-$K_{0}=\left[k_{0}^{\omega -1};\cdots ;k_{0}^{1};k_{0}^{0}\right].$  4:**for** i=1 to d−1 **do**  5:   **if** $[k_{i}^{w-1},\cdots k_{i}^{1},k_{i}^{0}]=0$ **then**  6:     st1: recoding-$K_{i}$ = recoding-$K_{i-1}$  7:     st2: S[i]=0  8:   **else**  9:     st1: recoding-$K_{i}$ = $K_{i}$10:     st2: S[i]=111:   **end if**12:**end for**13:**return** recoding-*k*,*S*.

In the ECSM algorithm, the recoding-k algorithm runs before each comb main cycle and only operates on the input scalar k, which takes up few circuit area resources. In the comb algorithm, time is calculated in “running time of modular multiplication”. The recoding-k algorithm will result in a certain time loss, but it is much less than a modular multiplication time.

### 3.4. ECPD and ECPA

Considering the storage burden of the pre-calculation, this paper uses affine coordinate to store the pre-calculation results. At the same time, the mixed operation of affine coordinate and Jacobi coordinate in ECPA operation can effectively reduce the calculation steps and reduce the calculation burden, and balance the shortage of the increase in the calculation amount in the multiple ECSM operation.

In the architecture implemented in this article, ECPA and ECPD will be carried out jointly in order to maintain consistency of operations in each loop. Therefore, ECPA and ECPD can be interwoven to reduce the number of operations. During the actual ECSM operation, the branches in the comb algorithm are actually replaced by sequential execution as a result of the recoding-k algorithm. That is, ECPD and ECPA are executed once per loop. So we could combine ECPD and ECPA. The specific ECPDPA algorithm is shown in Algorithm 3. At the same time, consider that the pre-calculation process also needs to use ECPD and ECPA, and ECPD and ECPA need to be calculated separately. Therefore, the circuit executed by the combination of ECPD and ECPA in this paper should also be used to calculate ECPD and ECPA separately, so as to improve the circuit reuse rate and reduce the circuit area.
**Algorithm 3** ECPDPA algorithm**Require: **$P=\left(X_{1}:Y_{1}:Z_{1}\right),Q=\left(x_{1}:y_{1}\right).$**Ensure: **$2P+Q=(X_{3}:Y_{3}:Z_{3})$  1:$T_{1}\leftarrow Z_{1}^{2}$  2:$T_{2}\leftarrow T_{1}\cdot Z_{1}$  3:$T_{1}\leftarrow T_{1}\cdot x_{2}$  4:$T_{2}\leftarrow T_{2}\cdot y_{2}$  5:$T_{1}\leftarrow T_{1}-X_{1}$  6:$T_{2}\leftarrow T_{2}-Y_{1}$  7:$Z_{3}\leftarrow Z_{1}\cdot T_{1}$  8:$T_{3}\leftarrow T_{1}^{2}$  9:$T_{4}\leftarrow T_{3}\cdot T_{1}$10:$T_{3}\leftarrow T_{3}\cdot X_{1}$11:$T_{1}\leftarrow 2T_{3}$12:$X_{3}\leftarrow T_{2}^{2}$13:$X_{3}\leftarrow X_{3}-T_{1}$14:$X_{3}\leftarrow X_{3}-T_{4}$15:$T_{3}\leftarrow T_{3}-X_{3}$16:$T_{3}\leftarrow T_{3}\cdot T_{2}$17:$T_{4}\leftarrow T_{4}\cdot Y_{1}$18:$Y_{3}\leftarrow T_{3}-T_{4}$**Return** $2P+Q=(X_{3}:Y_{3}:Z_{3})$.

### 3.5. Field Algorithm

In the ECSM computing framework, ECPA and ECPD scheduling realize ECSM operation, and field computing scheduling realizes ECPA and ECPD. In this paper, we use the interleaved modular multiplication algorithm, binary inverse algorithm, and the advance carry adder to realize the field calculation.

The modular multiplication algorithm is shown in Algorithm 4. This article designs modular multiplication units based on the interleaved modular multiplication algorithm. For standard interleaved modular multiplication algorithms, the modular reduction of intermediate results usually requires a maximum of two comparison and subtraction operations. We pre-calculate the second subtraction operation to fix the computation time of the modular multiplication unit.

The modular inverse algorithm is shown in Algorithm 5. The binary inverse algorithm uses simple shift and subtraction operations, replacing the complex division operations in the extended Euclidean algorithm. Furthermore, the algorithm reduces the length of the addition chain by adopting a parallel pre-calculation method. This optimization strategy not only shortens the data path, but also avoids increasing the cycle of modular inversion operations.
**Algorithm 4** Interleaved modular multiplication algorithm**Require: **Integera,b∈[0,p−1],primep.**Ensure: **c=(a×b)modp  1:c=0  2:$p^{\prime }=2\times p$  3:**for** i=n−1 to 0 **do**  4:   $c_{1}=2\times c$  5:   t=a[i]×b  6:   $c_{2}=c_{1}+t$  7:   $c_{3}=c_{2}-p$  8:   $c_{4}=c_{2}-p^{\prime }$  9:   **if** $c_{2}⩾p^{\prime }$ **then**10:     $c=c_{4}$11:   **else if** $c_{2}⩾p$ **then**12:     $c=c_{3}$13:   **else**14:     $c=c_{2}$15:   **end if**16:**end for**17:**return** c=(a×b)modp.
**Algorithm 5** Modular inverse algorithm**Require: **Integera∈[0,p−1],primep.**Ensure: **$c=a^{-1}\;mod\;p$  1:u=a,v=p,x=1,y=0  2:**while** u≠1andν≠1 **do**  3:   **while** u[0]=0 **do**  4:     u=u/2  5:     **if** x[0]=0 **then**  6:        x=x/2  7:     **else**  8:        x=(x+p)/2  9:     **end if**10:   **end while**11:   **while** v[0]=0 **do**12:     v=v/213:     **if** y[0]=0 **then**14:        y=y/215:     **else**16:        y=(y+p)/217:     **end if**18:   **end while**19:   **if** u⩾v **then**20:     u=u−v,x=(x−y)modp21:   **else**22:     v=v−u,y=(y−x)modp23:   **end if**24:**end while**25:**if** u=1 **then**26:   c=xmodp27:**else**28:   c=ymodp29:**end if**30:**return** $c=a^{-1}\;mod\;p.$

## 4. Data Dependency and Timing Analysis

**Figure 2:**
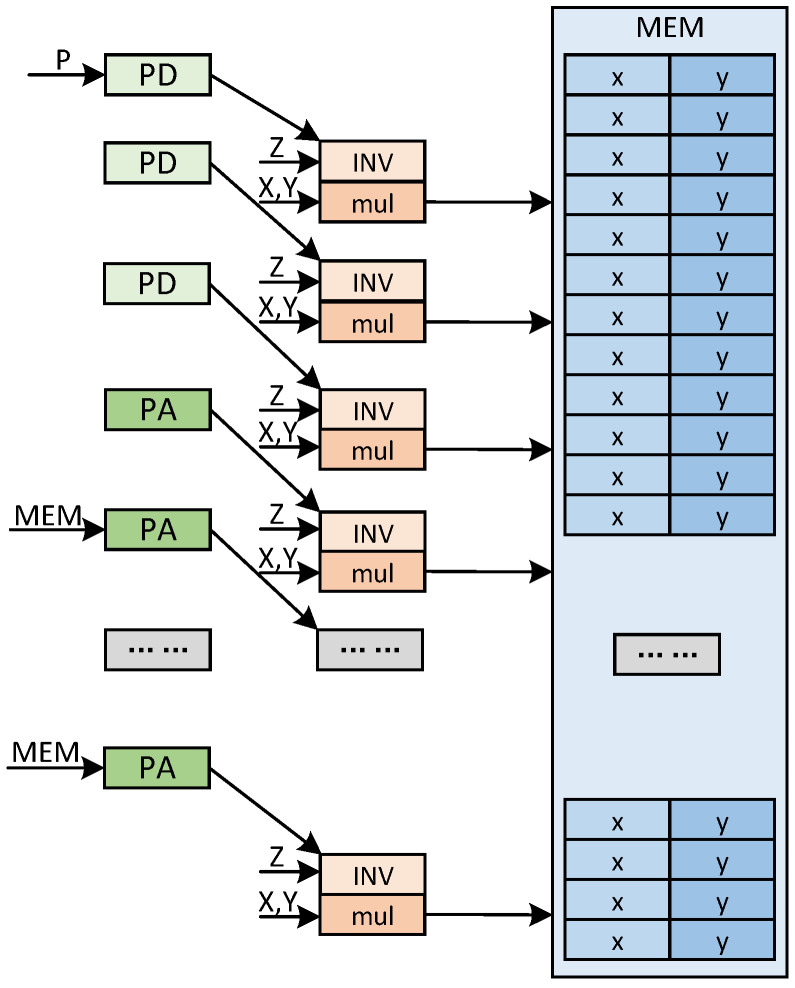
Date dependence and stream of pre-calculation in different modules.

Firstly, the clock cycles of the pre-calculation phase are calculated. The scheduling of the pre-calculation module in the ALU is shown in Figure 2. In the scheduling of the pre-calculation module, the data dependency of the pre-calculation stage is shown. First, the ECPD or ECPA calculation is performed. After the calculation, the result is entered into the next level of calculation flow. At the same time, the calculation results are put into the modular inverse operation, and the z-coordinate is inverse. And then the XY coordinates are restored. The result of the restoration is stored in the storage module.

It can be seen that only the last modular inverse operation cannot be run at the same time as ECPA or ECPD, so only the operation time of one modular inverse needs to be considered. The ECPA or ECPD itself and the ALU are computed via modular multiplication separately. In coordinate reduction, consider the reduction of Jacobi coordinate to affine coordinate. With reasonable arrangement of the operation results, just 4 times modular multiplication can achieve the restoration of affine coordinate.

As mentioned above, the total calculation clock cycles during the pre-calculation phase is

(13) $$\begin{array}{c} CC_{PRE}=64\times CC_{ECPD}+2\times (64\times CC_{ECPD}+4\times \\ CC_{MM})+11\times \left(CC_{ECPA}+4\times CC_{MM}\right)+CC_{INV} \end{array}$$

where $CC_{PRE}$ refers to the total clock cycles of the pre-calculation operation and $CC_{ECPD}$, $CC_{ECPA}$, $CC_{MM}$, and $CC_{INV}$ refer to the clock cycles of ECPD operation, ECPA operation, single modular multiplication operation, and modular inversion operation, respectively. Given that

(14) $$\left\{\begin{array}{c} CC_{ECPD}=4\times CC_{MM}\\ CC_{ECPA}=5\times CC_{MM} \end{array}\right.$$

$CC_{PRE}$ can be written as

(15) $$CC_{PRE}=875\times CC_{MM}+CC_{INV}$$

**Figure 3:**
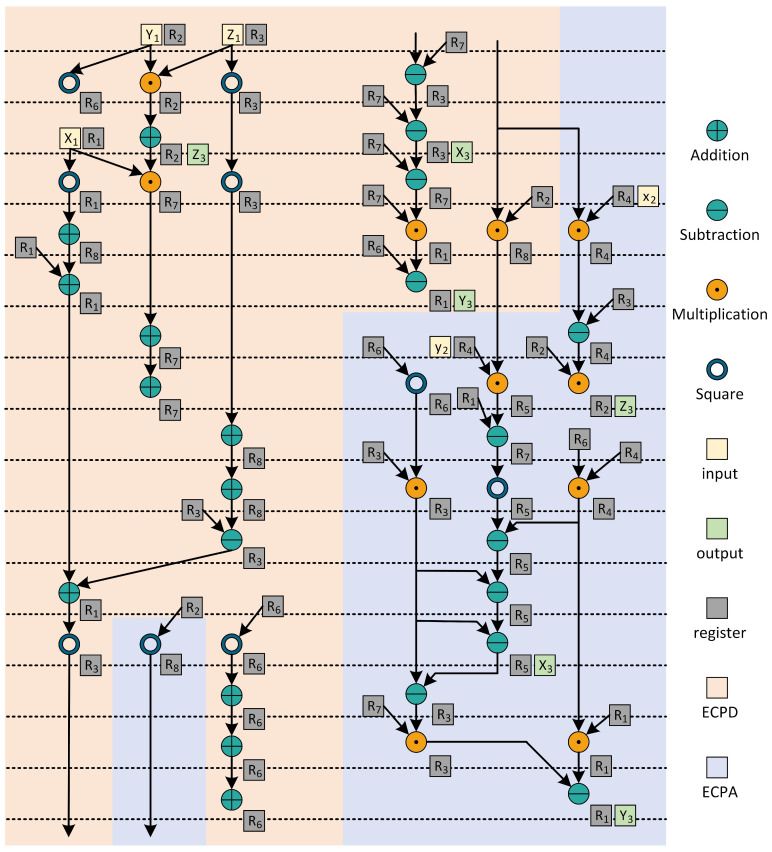
Hardware finite field operation scheduling diagram of the overall ALU of ECPDPA with ECPD and ECPA inside.

Next, the clock cycles consumed by the comb main cycle are calculated. In the comb main cycle, the data flow and scheduling of field calculation are shown in Figure 3. The combined calculation of ECPD and ECPA is realized by using eight registers. In this paper, the modular addition unit and modular subtraction unit are realized by a combination circuit. Therefore, the clock cycles of a comb are $7\times CC_{MM}$. The scalar *k*, which is 256 bits wide, runs 63 rounds of comb operations during comb computation. Therefore, a total of $63\times 7\times CC_{MM}=441\times CC_{MM}$ clock cycles are consumed in the comb main cycle.

(16) $$CC_{COMB}=441\times CC_{MM}$$

Finally, the clock cycles consumed by the coordinate restoration are calculated. The last part of the whole ECSM algorithm process is coordinate restoration. As with the pre-calculated coordinate restoration method, the coordinate restoration after the end of the main cycle also requires one modular inverse operation and four modular multiplication operations.

(17) $$CC_{POST}=4\times CC_{MM}+CC_{INV}$$

## 5. The Hardware Architecture

### 5.1. Overall ECSM Architecture

**Figure 4:**
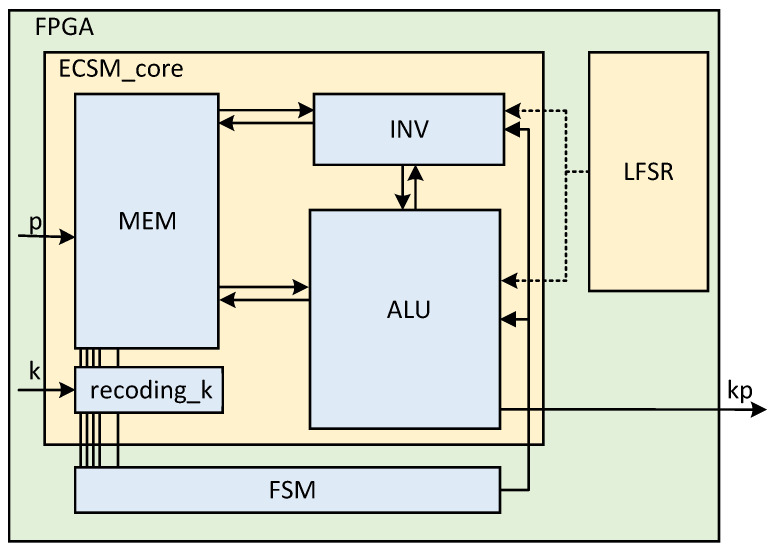
Overall ECSM architecture on FPGA and top input/output.

The overall system architecture of ECSM is shown in Figure 4. It consists of an ALU, a modular inverse module, a recoding-k module, and a pre-calculated storage register group (MEM). The code generated by the external controller controls the overall ECSM operation. The external linear feedback shift register (LFSR) provides pseudorandom numbers that provide input to the randomization-Z algorithm and act as the Z coordinate for randomization. The same input can produce different intermediate results, while the real results remain the same. In this way, the security of algorithm implementation is improved.

**Figure 5:**
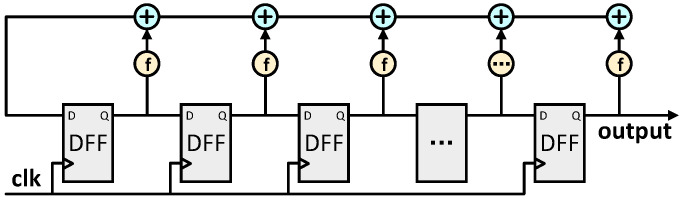
Circuit structure diagram of Fibonacci linear feedback shift register.

The LFSR is an algorithm for generating sequences of pseudorandom numbers. When generating a 256-bit pseudorandom number, an initial seed is used. And then each bit of output is iteratively generated through linear feedback. This is shown in Figure 5. Specifically, the LFSR consists of multiple register bits and a specific feedback polynomial. In this way, the output of each bit of the LFSR depends not only on the value of the current bit, but also on the value of the previous bits. At each iteration, the current state of the register is updated according to this polynomial, and the result is fed back to the input side of the register.

Although the proper selection of the initial LFSR seed can provide good randomness, the pseudorandom numbers generated by the LFSR are ultimately circular. Therefore, in the design of this paper, the random number input port is led to the top layer of the module. The LFSR can be replaced by a true random number generator (TRNG) in practical applications. Through a TRNG, true Z-coordinate randomization is realized.

According to Algorithm 2, the hardware architecture of recoding-k is realized. Here, the input register, control logic, comparator, multiplexer, and output register are included. The input register is used to store input parameters. The control logic includes a loop counter and a state machine that schedules the execution of the entire algorithm. The comparator generates a selection signal. The multiplexer selects the appropriate input data for the operation to update the value of the S-array. The final result is stored in the output register and output.

According to Algorithm 1, the overall system architecture of ECSM works as follows. The working state determines how the ALU is calculated. The ALU obtains data from the INV, MEM, or its own feedback loop. After the ALU completes the calculation, the calculation results have three directions according to the different working states. They are stored in the MEM, passed to the INV for computation, or passed to the ALU internal loop. The INV always processes the data from the ALU and sends the results back to the ALU to participate in the computation. The MEM stores the results of the pre-calculated calculation, and passes the corresponding data to the ALU to participate in the calculation according to the results generated by scanning recoding-k.

In terms of flexibility, the architecture proposed in this paper pays special attention to parametric implementation. This design method allows the system to flexibly adapt to a variety of requirements. Through parameterization, the proposed architecture can be compatible with different prime numbers. Furthermore, when dealing with a larger field, parametric configuration is also adopted in this paper. This means that the architecture can be easily reconfigured on demand to become an arbitrarily bit-wide ECSM architecture. For example, the common GF(384) and GF(521).

### 5.2. Arithmetic Logical Unit

**Figure 6:**
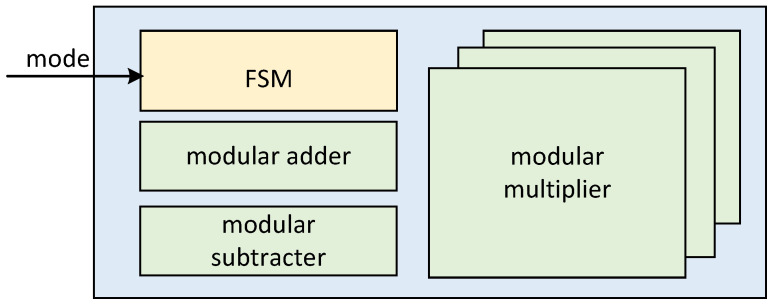
Inner architecture of ALU and different internal modules.

Considering the overall architecture of ECSM implemented in this paper, we propose a general-purpose arithmetic logical unit (ALU), through which the joint implementation of ECPA and ECPD in the comb process can be realized. The ALU consists of three parallel modular multipliers, a modular adder/subtracter and corresponding state machine control module. The ALU hardware architecture is shown in Figure 6.

With this ALU hardware architecture, we can be compatible with different working modes. As shown in Table 2, four working modes are realized. They are the joint implementation of ECPA and ECPD, the independent implementation of ECPA and ECPD, and the working mode of calculating modular multiplication separately.

**Figure 7:**
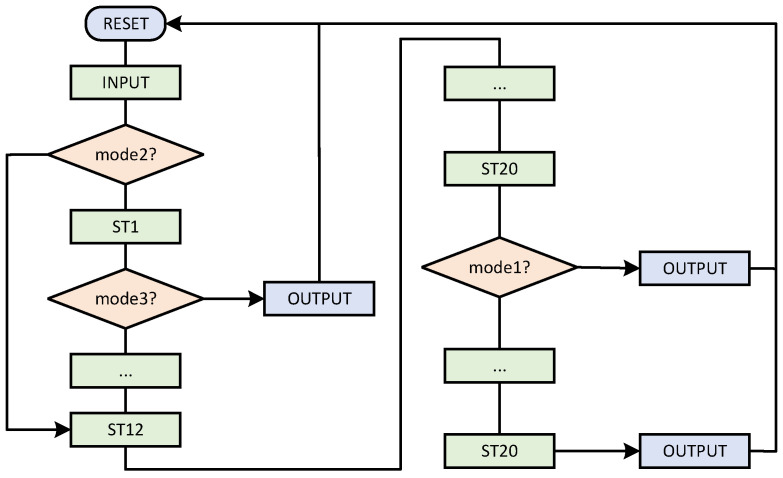
Jump flow chart of state machine in ALU in different modes.

The different working modes are described in detail next. The jump flow chart in different modes is shown in Figure 7.

Mode0 is a joint implementation of ECPA and ECPD. *P* coordinates and *Q* coordinates are input to achieve 2P+Q output according to the ECPA mixed-coordinate formula. Mode1 is an ECPD independent implementation that implements 2P output based on the input *P* coordinate. Mode2 is an independent implementation of ECPA, based on the input *P* coordinate and *Q* coordinate, to achieve P+Q output. Mode3 is a separate calculation of modular multiplication, according to the input a,b,p to achieve a×bmodp output.

Different modes of operation play different roles in the overall ECSM. Mode0 is the mode executed in the comb main cycle to speed up the main cycle and reduce clock consumption. Since ECPD and ECPA must alternate in the main cycle, ECPD and ECPA are calculated jointly. Therefore, reasonable scheduling of data flow can save time. Mode1 and mode2 are executed in the pre-calculation. In the pre-calculation, mode1 and mode2 store the result in the appropriate register. Since the number of ECPDs in the calculation is much more than that of ECPA, it is more time saving to calculate ECPD and ECPA separately. Mode3 is used in coordinate conversion. The need for coordinate transformation is generated only when the calculation is pre-calculated and the result is output. Therefore, it is efficient to set up compatible operating modes and thus reuse circuits.

**Figure 8:**
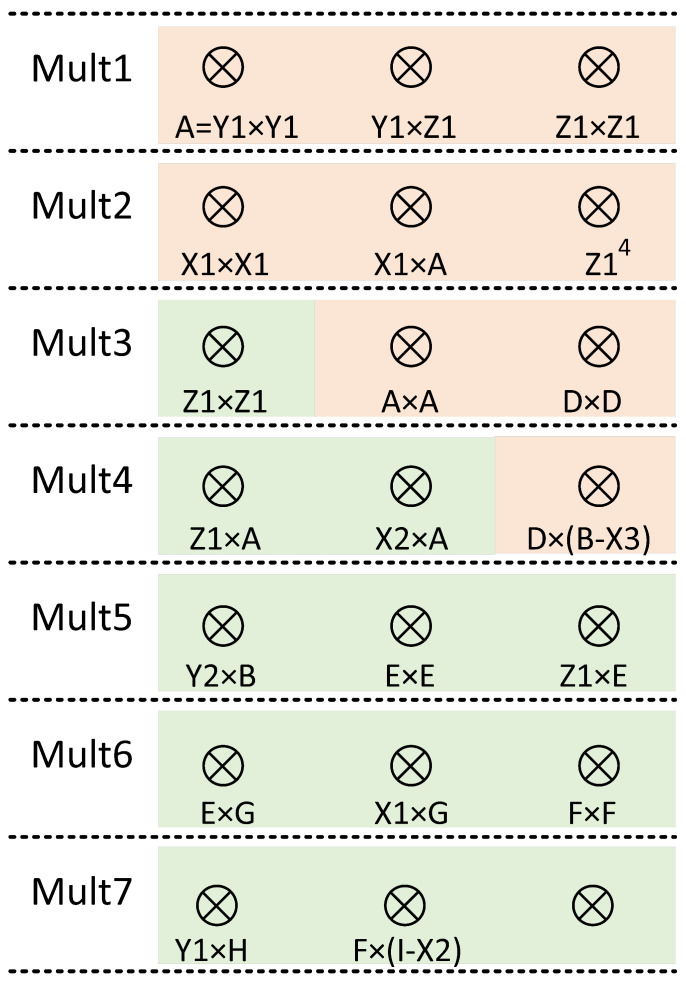
Scheduling of three multipliers in ALU in which the yellow background is ECPD and the green background is ECPA.

The calculation time of modular addition and subtraction in ECSM is much less than that of modular multiplication. And the modular multiplication implemented in this paper is a modular multiplication unit with constant time. So it is reasonable to use parallel modular multiplication times to calculate the operation time, which is shown in Table 3. The scheduling relationship of three parallel multipliers in the ALU is shown in Figure 8. Through reasonable scheduling of the data relationship between multipliers, ECPDPA calculation can only consume 7 times modular multiplication, and ECPD alone requires 4 times modular multiplication, and ECPA alone requires 5 times modular multiplication. ECPDPA takes 7 < 4 + 5, saving 2 times modular multiplication.

### 5.3. Pre-Calculation

In the comb algorithm, a pre-calculation operation is required. The calculated results are stored in the register bank. In each comb cycle, the coordinate under the corresponding address are read based on the scanned bits by the ALU. In this article, ECSM is considered for computing a 256-bit-wide scalar *k*, where ω=4 in the comb algorithm. Therefore, it is necessary to traverse $\left(2^{4}-1\right)$ combinations in the pre-calculation. Specifically, it is necessary to perform three consecutive 64 times ECPD. Then the results are arranged separately. That is, using ECPA operations, all the results that need pre-calculation are iterated through. This is a scheme that consumes too much area to implement the pre-calculation circuit alone. Considering this, this paper adopts a universal ALU, compatible with ECPD and ECPA, which greatly reduces the circuit area.

### 5.4. Modular Operation

The architecture implemented in this paper includes three modular operations, namely modular multiplication, modular addition/subtraction, and modular inversion.

**Figure 9:**
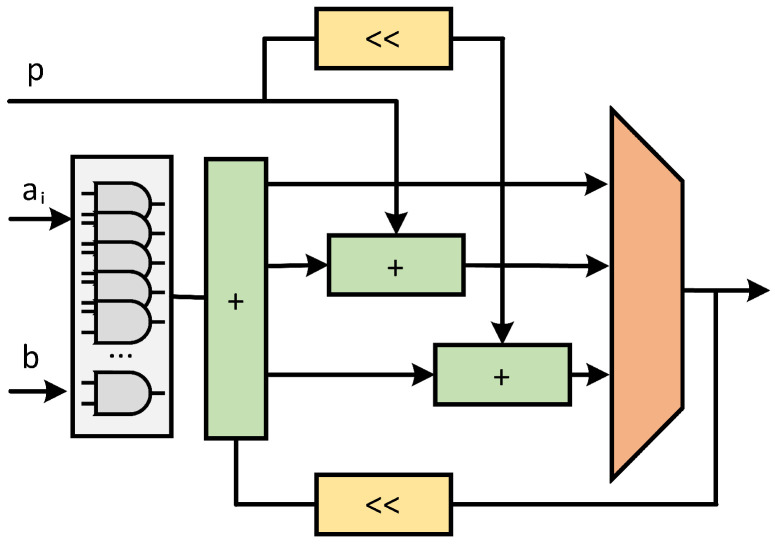
Block diagram of interleaved modular multiplication algorithm. In this figure, the same color means the same function.

The modular multiplication is implemented according to Algorithm 4. The specific structural block diagram of interleaved modular multiplication algorithm is shown in Figure 9. By adding a single subtracter, the original serial subtraction operation is converted into parallel operation in this design. In this way, the purpose of pre-calculation processing is achieved. The critical path elongation is reduced. The calculation time of each modular multiplication operation is guaranteed to be fixed. In this way, the balance between circuit area and calculation time is realized.

**Figure 10:**
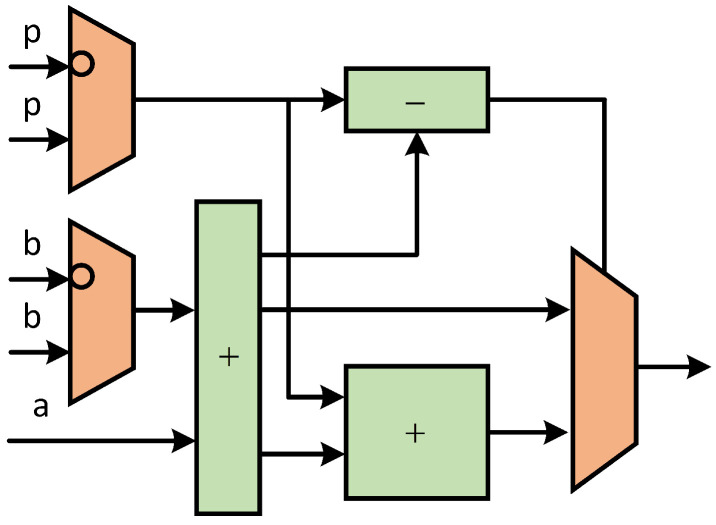
Block diagram of modular addition/subtraction. In this figure, the same color means the same function.

The modular addition/subtraction is implemented integrally. The block diagram of the modular addition/subtraction unit is shown in Figure 10. The conventional addition operation of the input values *a* and *b* is carried out by the adder circuit. The modular *p* operation is realized through the subtracter circuit and the comparator circuit. In this process, the subtracter circuit is always used to perform the subtraction operation between the output result of the adder circuit and the value of *p*. Thus, the function of pre-calculation can be realized. The comparator circuit is used to judge the relationship between the output result of the adder circuit and the value of *p*. According to the judgment result of the comparator circuit, the output result of the adder circuit or the output result of the subtracter circuit is finally selected as the final output.

The modular inversion is implemented according to Algorithm 5, and the detailed architectures are described next. The binary inversion algorithm uses the shift and subtraction operation which is easily realized by FPGA, and the complex division operation is realized. In the prime number field, the initial value x=1 is modified to x=b to realize the operation of b/amodp, and the operation time consumption is basically the same as the original binary inverse algorithm.

**Figure 11:**
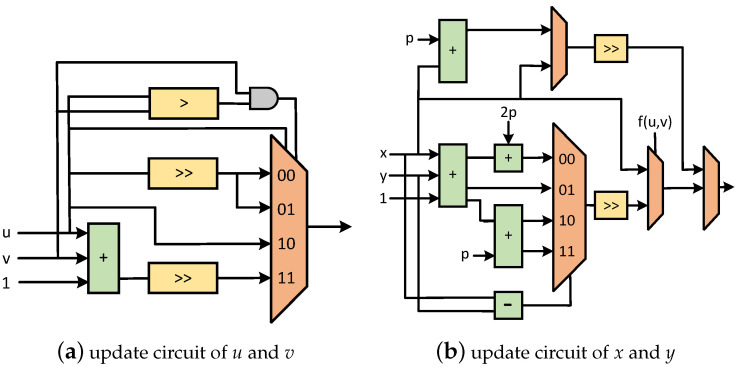
Block diagram of critical registers that need to be iterated in binary inversion algorithm. In this figure, the same color means the same function.

The block diagram of the binary inversion algorithm is shown in Figure 11. Figure 11a is the updated computing circuit for *u* and *v*. Figure 11b is the updated computing circuit for *x* and *y*. By calculating the updates of *u* and *v*, the updates of *x* and *y* can be achieved. In order to reduce the length of the critical path, we divide the original addition chain into three parallel additions. Through the method of pre-calculation, the modular inversion improves the calculation speed and realizes the balance between circuit area and calculation time.

## 6. Result Analysis and Comparison

In this section, the ECSM framework implemented in this article is evaluated. Firstly, the common indexes used in evaluation are introduced. Then, according to the indicators, the ECSM implemented in this paper is evaluated and compared with the results of other scholars.

The evaluation indicators and methods adopted in this paper are described as follows:

1. Delay and performance analysis: Data latency and performance are the basis for evaluating ECSM. An efficient ECC implementation ensures that devices remain responsive and efficient when handling encryption operations. In this paper, the calculation is based on the clock cycles consumed by the ECSM calculation and the ultimate realized maximum frequency of the system.
  (18) $$time=\frac{CC_{total}}{f_{max}}$$
2. Area-time product: Considering the application scenarios of ECSM, how to save resources also needs to be considered. ATP metrics help in selecting the implementation that provides the best performance whilst saving limited hardware resources. The tradeoff between resource footprint and data latency is often evaluated using the area-time product. In this paper, the consumption of equivalent slice resources is used to evaluate the area. Specific equivalent methods are described below.
  (19) ATP=slice×time
3. Throughput per Slice In an ECSM implementation, throughput represents the overall architecture’s ability to process data per unit of time, directly affecting its performance and efficiency. It is estimated here as a ratio of the processing bit width to the total time consumed, as shown below.
  (20) $$Throughput\left(TP\right)=\frac{Bit\;Width}{time}\;bps.$$
  To evaluate the tradeoff between hardware resource consumption and throughput, we use the ratio of throughput to area as performance.
  (21) $$Performance\left(Perf\right)=\frac{Throughput}{slices}\times 10^{3}.$$
4. Resistance of side-channel attack: If the ECSM side-channel attack is not secure enough, the security of the whole ECC system will be seriously threatened.

### 6.1. Comparison between Pre-Calculation and No Pre-Calculation

Considering that in the ECDSA algorithm, the bit width of elliptic curve base point and scalar k is set based on the public standard, pre-calculation based on the elliptic curve base point and scalar k is safe and feasible. Moreover, pre-calculations do not need to be performed before every ECSM run. If the base point of the elliptic curve does not change, then the ECSM does not need to perform repeated pre-calculation. And that is exactly what happened. In the ECSM architecture designed in this paper, the circuit is compatible with pre-calculation. That is, the total area of the circuit is constant whether or not pre-calculation is performed. There is no additional circuit area cost due to the need for pre-calculation. According to the previous timing analysis chapter,

(22) $$CC_{PRE}=875\times CC_{MM}+CC_{INV}$$

(23) $$CC_{COMB}=441\times CC_{MM}$$

(24) $$CC_{POST}=4\times CC_{MM}+CC_{INV}$$

Therefore, the total time cost of the ECSM architecture designed in this paper is

(25) $$CC_{total}=\frac{CC_{PRE}+n\times \left(CC_{COMB}+CC_{POST}\right)}{n}$$

(26) $$CC_{total}=\frac{\left(875+445n\right)\times CC_{MM}+\left(1+n\right)CC_{INV}}{n}$$

where *n* refers to the total number of ECSM operations required after the pre-calculation.

**Figure 12:**
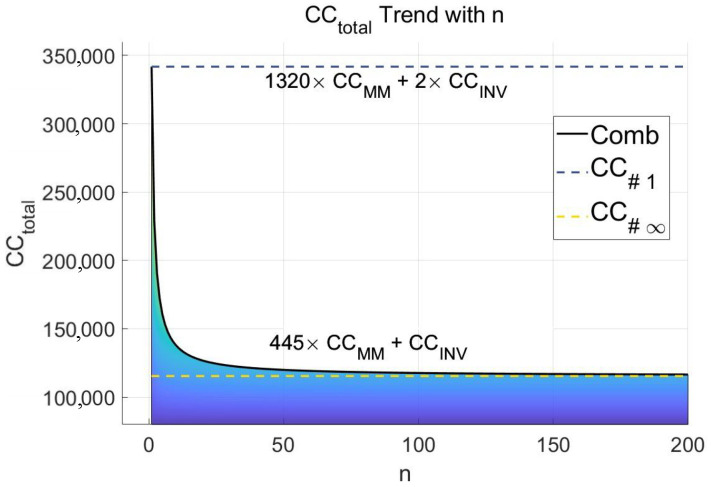
The upper and lower limits of $CC_{total}$ and the trend of change with *n*.

The trend in $CC_{total}$ is shown in Figure 12. With the increase in *n*, the burden of pre-calculation is gradually eliminated, and $CC_{total}$ is continuously reduced. The case where n=1 is the heaviest projected burden is called $CC_{\#1}\;$, and the case where n=∞ shares the projected burden after a large number of ECSM calculations is called $CC_{\#\infty }$

(27) $$CC_{\#1}=1320\times CC_{MM}+2\times CC_{INV}$$

(28) $$CC_{\#\infty }=445\times CC_{MM}+CC_{INV}$$

### 6.2. Security Analysis

In the ECSM architecture implemented in this paper, there is good resistance to side-channel attacks. In the process of scanning the input scalar k, because the scanned bits may be all zeros, there is a potential side-channel attack. In order to solve this problem, this paper adopts the recoding-k algorithm to avoid this possibility. The process of the recoding-k algorithm itself is also symmetrical, and there is no possibility of a side-channel attack. In addition, the modular multiplier implemented in this paper also has time constancy, avoiding the observable leakage of data in a large number of modular multiplier operations. Therefore, the ECSM architecture implemented in this paper is secure against SCA.

### 6.3. Result Comparison

**Figure 13:**
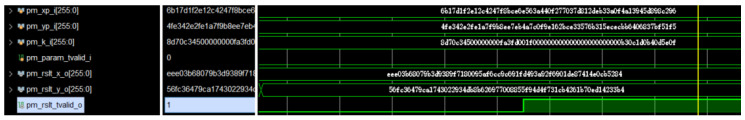
The simulation diagram related to FPGA implementation. In this figure, the correct data can be read by the yellow line.

To make a fair comparison with existing work, we implemented the proposed architecture using Vivado 2022 on Xilinx Virtex-7 series FPGA. According to the parameters recommended by the NIST standard [16], the ECSM calculation is carried out for the 256-bit scalar k. In general, there are five field operation units included in this design. They are one modular inverse unit, three modular multiplication units, and one modular addition/subtraction unit. In the architecture implemented in this paper, the modular multiplier is implemented with a fixed duration strategy and $CC_{MM}$ is 258. The average computing time of the modular inversion $CC_{INV}$ is 600. So the performance index of $CC_{\#1}=341,760$, $CC_{\#\infty }=116,010$ is obtained. The hardware area and maximum operating frequency of the ECSM architecture are given by Vivado 2022. And the simulation diagram related to FPGA implementation is shown in Figure 13.

Under normal circumstances, it is expected to run continuous ECSM after the pre-calculation. In this case, the architecture proposed in this paper occupies 116,017 clock cycles. The overall architecture has a maximum operating frequency of 157.7 MHz. One ECSM is calculated to cost 0.74 ms. No DSP computing resources or BRAM storage resources are used. The overall architecture consumes only 25,103 LUT resources, occupying 7351 slices. The ATP calculated on this basis is 5.41. In the worst-case scenario, where ECSM is expected to run only once after the pre-computation, the architecture proposed in this paper occupies 341,760 clock cycles. One calculation of ECSM costs 2.17 ms. Area consumption does not increase. The ATP calculated on this basis is 15.93.

Table 4 shows a comparison of the ECSM architecture proposed in this paper and related results. It should be noted that $this\;work_{\#1}$ and $this\;work_{\#\infty }$ are different implementations of the same architecture. $this\;work_{\#1}$ is the worst-case implementation of the architecture proposed in this article; however, this outcome is very rare. $this\;work_{\#\infty }$ is the result of the implementation of the proposed architecture under appropriate conditions, and it is also very close to the actual use case.

Now-common ECC encryptions, for example, the NIST standard [16], use 256-bit-wide scalar *k* for ECSM. The following comparisons all consider computing a scalar *k* with a 256-bit width. The particular bit width of the particular curve will be specified later in the description. Except where otherwise noted, all comparison references implement the ECSM architecture on Virtex-7 series FPGA. Compared with [37,38], this paper puts forward the ECSM architecture on the ECSM computing time and resource consumption indicators are dominant. Compared with [40], although the ECSM implemented in this paper takes a slightly longer computation time, the slices resource occupies only one-third.

In [41], the author does not give the specific slice resource usage. Generally, during the implementation of FGPA, each slice takes around 3.5 to 4 LUTs, and we call this ratio LUT/slice. This is estimated using LUT/slice=4. In the following comparison, LUT/slice=4, which is unfavorable to us, is used to convert LUT resources to slice resources. It can be seen that, in the worst case, $this\;work_{\#1}$ is slightly worse than [41]. However, with the increase in calculation times, the time delay of this design decreases rapidly. In common cases, $this\;work_{\#\infty }$ has a significant time advantage over [41]. For [33,40,42,43,44], with similar area occupation, the time advantage of this paper is obvious. In the ECSM architecture proposed by Hu [43], although the area occupation is low, due to the maximum frequency limitation and the increase in the number of calculation cycles, the operation time is very long, and the ATP increases rapidly. The architecture proposed by Hao et al. [50] performs better than $this\;work_{\#1}$, but not as well as $this\;work_{\#\infty }$. In the case of similar area occupation, the calculation time of $this\;work_{\#\infty }$ is shortened by 56.5%.

Including the regular LUT and slice resources, many ECSM framework designs also use additional DSP or BRAM resources to accelerate computing. Therefore, in order to make a fair comparison with the existing relevant work, this paper converts different indicators into equivalent slice numbers. According to the document [40], each V-4 DSP block has 619 slices, each V-5 DSP block has 992 slices, and each V-7 DSP block has 1475 slices. According to the document [40], each BRAM is equivalent to 281 slices. The reference [52] also focuses on the cost of equalizing ECSM computation time and area consumption. With the use of DSP and BRAM resources, the area consumption is large. In contrast, this paper has obtained certain advantages in both area and time. For references [45,46] and reference [11], design 1 and design 2 both need a large number of DSPs. Although they obtained a remarkable time advantage, the circuit area of consumption is larger. Some ECSM architectures base their designs on specific elliptic curves. The references [47,49] are designed based on Curve 448. The scalar bit widths are 224-bit and 448-bit, respectively. Compared to reference [49], $this\;work_{\#\infty }$ only occupies 56.6% of the area, and the calculation time is increased by 35.7%. The throughput per slice of this design is also improved a lot. For reference [47], although the calculation time of this design is not dominant, slice resources only occupy 5% of [47]. In this case, ATP in this paper still has the advantage. The throughput per slice of this design is also improved a lot.

In addition to the performance advantages, the ECSM architecture implemented in this paper is also resistant to side-channel attack. In contrast, not all references consider safety. In view of possible side-channel attacks, hardware defense is carried out accordingly in this architecture. In the main loop phase, the optimized comb-4 algorithm is used. For different input scalars, the unified calculation operation is realized. There is no difference in calculation operations due to differences in input scalars. Thus, the possible hidden trouble of sample power analysis is avoided. In the pre-calculation phase, the recoding-k algorithm is used. A small number of symbol bits are added to re-encode the input scalar. This avoids the possibility of scanning to a zero value and overcomes the potential for zero analysis attacks. The recoding-k algorithm itself also has operational consistency, avoiding sample power analysis. Neither th erecoding-k algorithm nor the unified computation operations in the main loop have redundant operations, thus increasing the security against fault injection attack. In FPGA implementation, the randomization-Z algorithm is added. The input scalar is randomized by the random number method. Thus, the ability to resist differential power analysis is improved.

Some ECSM architectures are based on improved multipliers. Compared to references [48,51], the ECSM architecture implemented in this paper is more secure, although the performance indicators in this paper are not superior to the above references. In the hardware scheduling of references [48,51], inconsistent operations are generated due to different scanned scalar bits. Specifically, the multiplier produces a selection during scanning due to the different scalar bits scanned, which are not uniform. The multiplier may be assigned from the previous register, or it may be zeroed out directly. The architecture designed in this article avoids the SCA risk that arises from this operational inconsistency and is therefore more secure. In addition, the randomization-Z algorithm is added to further improve the security in this paper, which is not available for references [48,51].

We also employed the Xilinx platform to estimate the power consumption of the proposed design. The power consumption of the architecture implemented in this paper is 413 mW during operation. Since most references in Table 4 do not provide power data, power comparison is performed separately in Table 5. Through comparison, it can be found that the ECSM architecture implemented in this paper has a higher performance and frequency, so it has a higher power consumption. As mentioned earlier, the ECSM architecture implemented in this paper is more secure than that in reference [48], which brings a corresponding increase in power consumption. However, this design is suitable for high-performance applications with higher safety requirements, where the necessary cooling measures and sufficient power supply are equipped.

The ECSM architecture proposed in this paper has configurability. The bit width of the underlying finite field operation can be changed by modifying the preset parameters. In this way, the architecture can be compatible with ECSM calculations of any bit width. This includes safer large bit widths, such as GF(384) and GF(521). Due to the limited hardware resources inside the small-level FPGA platform, the deployment of a larger-scale ECSM architecture will lead to a rapid increase in congestion level. This results in a significant decrease in the overall operating frequency of ECSM. Therefore, for large finite-field ECSM calculations, larger FPGA are needed. For example, Virtex Ultrascale series FPGA. GF(384) has a maximum operating frequency of 129.3 MHz. GF(521) has a maximum operating frequency of 110.9 MHz.

Since the underlying operations of $GF(2^{n})$ and GF(p) are different, the proposed architecture cannot directly compute ECSM in $GF(2^{n})$. But by changing only the underlying cell, the proposed architecture can easily migrate the top-level algorithm to $GF(2^{n})$.

Ultimately, this paper analyzes the possibility of common SCAs, increases the corresponding protection means, and realizes the resistance of side-channel attack. By adopting reasonable algorithm design and designing optimized hardware architecture and computing unit, this paper realizes speed improvement while taking into account resources so that ATP achieves significant advantages in the comparison of ECSM implementation results.

## 7. Conclusions

This article introduces a comb algorithm based on recoding-k. It is designed for computing scenarios with limited resources and high security requirements, while taking into account performance speed and circuit area. Considering common SCAs, the architecture proposed in this paper increases the security design, realizes the resistance of SCA, and improves the security of the algorithm implementation. In terms of computing speed and resource consumption, this paper proposes a compact multiplier scheduling arrangement based on the dependency relationship in ECSM data. In this way, the computing speed is improved and the resource occupancy is optimized. In this paper, we implement the ECSM architecture and optimize the hardware structure by using the above algorithms. This includes a recoding-k unit, a modular inversion unit, a memory array, a control unit, and an ALU. Based on the pre-calculation burden and the main loop calculation burden, the comb-4 algorithm is chosen. We have achieved resource reuse and maximum security for computing scenarios with area-time limitations. The implementation results show that the proposed architecture performs on par with common algorithms with comb-$\omega_{\#1}$. With comb-$\omega_{\#\infty }$, the ATP of this design is superior to the existing best works. In future work, we will seek improvements in this field, further reducing resources and power consumption, improving performance, and attempting to implement hardware architecture on an application-specific integrated circuit.

## Figures and Tables

**Table 1:** Comparison of the computational burden of different algorithms.

	ECPD	ECPA
double-and-add	*n* − 1	$\frac{n-1}{2}$
always double-and-add	*n* − 1	*n* − 1
non-adjacent form	*n* − 1	$\frac{n-1}{3}$
Montgomery Ladder	*n* − 1	*n* − 1
Joye’s double-add	*n* − 1	*n* − 1
Co-Z	$\left(n-1\right)\times \frac{8}{21}$	$\left(n-1\right)\times \frac{8}{21}$
comb-$\omega_{\#\infty }$	$\frac{n}{\omega }-1$	$\frac{n}{\omega }-1$
comb-$\omega_{\#1}$	n−1	$2^{\omega }-\omega +\left(\frac{n}{\omega }\right)-2$

**Table 2:** Inputs and outputs of different arithmetic logical unit modes.

Mode		0	1	2	3
input	*P*(*X*, *Y*, *Z*)	✓	✓	×	a,b,p
	*Q*(*x*, *y*)	✓	×	✓	
output		2*P* + *Q*	2*P*	*P* + *Q*	a×bmodp

**Table 3:** Modular multiplication times of different ALU modes.

FSM Stage	Operation	Times
stage1–stage30	ECPDPA	7
stage1–stage20	ECPD	4
stage12–stage30	ECPA	5
stage1	MM	1

In the table, MM refers to the modular multiplication operation.

**Table 4:** Implementation result comparison of FPGA.

	LUT	DSP	BRAM	Slice	Equival–Slice	Freq/MHz	CC	Latency/ms	ATP	Perf
$this\;work_{\#1}$	25,103	0	0	7351	7351	157.7	341,760	2.17	15.93	16.05
$this\;work_{\#\infty }$	25,103	0	0	7351	7351	157.7	116,010	0.74	5.41	47.06
[37]	–	0	0	11,300	11,300	121.5	–	3.27	36.95	6.93
[38]	–	0	0	8873	8873	177.7	262,650	1.48	13.13	19.49
[39]	65,600	0	0	22,000	22,000	327.0	–	0.47	10.34	24.76
[40]	–	0	0	5466	5466	124.0	464,100	3.73	20.39	12.56
[41]	22,151	0	0	–	5538	95.2	191,815	2.01	11.13	23.00
[42]	–	0	0	5500	5500	122.8	300,000	2.44	13.44	19.08
[43]	10,673	0	0	2932	2932	53.5	610,030	11.41	33.45	7.65
[44]	24,705	0	0	7101	7101	187.0	199,410	1.07	7.57	33.69
[33]	–	0	0	6543	6543	104.0	198,715	1.91	12.50	20.48
[45]	96,867	2799	242	2291	28,469,011	72.9	215,880	0.14	401.28	0.06
[11] DESIGN1	45,500	560	0	12,710	557,811	125.0	–	0.46	256.59	1.00
[11] DESIGN2	46,900	560	0	14,010	557,811	125.0	–	0.25	139.45	1.84
[46]	22,736	136	15	6909	211,724	232.3	32,272	0.14	29.64	8.64
[47]	–	88	0	7666	137,466	245.0	49,375	0.20	27.49	8.15
[48]	18,100	0	0	6200	6200	195.0	137,000	0.70	4.36	58.77
[49]	50,143	0	0	–	12,536	325.0	372,742	1.15	14.42	31.08
[50]	21,176	0	0	6397	6397	158.7	270,000	1.70	10.88	23.54
[51]	9320	0	0	2960	2960	238.0	–	0.69	2.04	125.34

In the table, if slice data are given in the reference, equival–slice is calculated using the data given in the article; otherwise, equival–slice is calculated using the calculation method given herein. If a reference uses DSP or BRAM resources, the corresponding data comparison is divided into two lines. The first line is the result of the comparison without considering additional resources. The second line considers the comparison results of additional resources. Reference [37] is implemented based on K7 series FPGA; references [39,41] is implemented based on V6 series FPGA.

**Table 5:** Power result comparison.

	Freq/MHz	Perf	Power/mW
this work	157.7	47.06	413
[43]	53.5	7.65	38
[44]	187.0	33.69	210
[48]	195.0	58.77	190

## Data Availability

All data can be provided upon reasonable request to the corresponding author.

## Abbreviations

The following abbreviations are used in this manuscript:

ALU	arithmetic logical unit
ECDSA	elliptic curve digital signature algorithm
ECC	elliptic curve cryptography

ECDH	elliptic curve Diffie–Hellman
IDDMM	iterative digit–digit Montgomery multiplication
ECPA	elliptic curve point addition
ECPD	elliptic curve point doubling
ECPT	elliptic curve point triple
RNS	residue number systems
NAF	non-adjacent form
SCA	side-channel attack
LFSR	linear feedback shift register
TRNG	true random number generator
INV	modular inverse
MM	modular multiplication
ASIC	application-specific integrated circuit
GF	finite field or Galois field
Variable	
x,y	affine coordinate
X,Y,Z	Jacobian coordinate
Mathematical symbols	
GF(*p*)	elliptic curve prime field
